# Effects of multidisciplinary teamwork on lead times and patient flow in the emergency department: a longitudinal interventional cohort study

**DOI:** 10.1186/1757-7241-21-76

**Published:** 2013-11-01

**Authors:** Åsa Muntlin Athlin, Ulrica von Thiele Schwarz, Nasim Farrohknia

**Affiliations:** 1 Department of Medical Sciences, Uppsala University, Uppsala University Hospital, 751 85 Uppsala, Sweden; 2 Department of Public Health and Caring Sciences, Uppsala University, Box 564, 751 22 Uppsala, Sweden; 3 Department of Emergency Care, Uppsala University Hospital, 751 85 Uppsala, Sweden; 4 School of Nursing, University of, SA 5005 Adelaide, Australia; 5 Department of Psychology, Stockholm University, 106 91 Stockholm, Sweden; 6 Karolinska Institutet, Department of Learning, Informatics, Management and Ethics, Medical Management Centre (MMC), 171 77 Stockholm, Sweden; 7 Head of Emergency Department, Södersjukhuset, Södersjukhuset AB, Sjukhusbacken 10, 118 83 Stockholm, Sweden; 8 Department of Clinical Science and Education, Södersjukhuset, Karolinska Institutet, SE-171 77 Stockholm, Sweden

**Keywords:** Teamwork, Emergency department, Waiting times, Lead times, Patient flow, Patient safety, 4-hour target

## Abstract

**Background:**

Long waiting times for emergency care are claimed to be caused by overcrowded emergency departments and non-effective working routines. Teamwork has been suggested as a promising solution to these issues. The aim of the present study was to investigate the effects of teamwork in a Swedish emergency department on lead times and patient flow.

**Methods:**

The study was set in an emergency department of a university hospital where teamwork, a multi-professional team responsible for the whole care process for a group of patients, was introduced. The study has a longitudinal non-randomized intervention study design. Data were collected for five two-week periods during a period of 1.5 years. The first part of the data collection used an ABAB design whereby standard procedure (A) was altered weekly with teamwork (B). Then, three follow-ups were conducted. At last follow-up, teamwork was permanently implemented. The outcome measures were: number of patients handled within teamwork time, time to physician, total visit time and number of patients handled within the 4-hour target.

**Results:**

A total of 1,838 patient visits were studied. The effect on lead times was only evident at the last follow-up. Findings showed that the number of patients handled within teamwork time was almost equal between the different study periods. At the last follow-up, the median time to physician was significantly decreased by 11 minutes (p = 0.0005) compared to the control phase and the total visit time was significantly shorter at last follow-up compared to control phase (p = <0.0001; 39 minutes shorter on average). Finally, the 4-hour target was met in 71% in the last follow-up compared to 59% in the control phase (p = 0.0005).

**Conclusions:**

Teamwork seems to contribute to the quality improvement of emergency care in terms of small but significant decreases in lead times. However, although efficient work processes such as teamwork are necessary to ensure safe patient care, it is likely not sufficient for bringing about larger decreases in lead times or for meeting the 4-hour target in the emergency department.

## Background

In a report from the Institute of Medicine, the emergency department (ED) setting has been declared a high-risk area for the complexities of patient safety issues [1]. Long waiting times and limited access to emergency care are claimed to be caused by overcrowded EDs and non-effective working routines [2,3]. This, in turn, might be a threat to patient safety as it can potentially delay time to assessment and treatment as well as raise the number of patients leaving without being seen. In line with this, excessive total waiting time, along with time to physician, has been suggested as an important quality indicator in the ED.

In EDs around the world new concepts have been introduced to handle these difficulties, such as “the 4-hour target”. The purpose of the 4-hour target in emergency care is that patients should be treated and discharged (to home or to ward) within 4 hours [4]. To meet this goal, the organization and routines in the EDs have to be changed. There is an argument that simply adding hospital beds and doing quick fixes are not a sufficient solution to decreasing waiting times and over-crowding. To make quality improvement in terms of waiting times, there has to be a continuous focus on improvements to the different processes in the patient flow [5].

In recent years, introducing multi-professional teams as a way of improving the quality of healthcare has shown promising results [6-8]. Furthermore, a recent published systematic review has shown that teamwork is crucial for improvements to patient flow processes in the ED [9]. However, there are no standard solutions and therefore changes have to be adjusted to the specific context [10]. A *work team* could be defined as a group composed of two or more individuals who (a) exist to perform organizationally relevant tasks, (b) share one or more common goals, (c) interact socially and (d) exhibit task interdependencies (i.e., workflow, goals, and outcomes) [11,12]. However, unpredictable and time-pressured work settings like the ED place extra demands on teams’ processes to achieve efficiency and team goals [7,12]. Although teamwork is encouraged to improve the EDs’ efficiency there seems to be a very limited number of published evaluations of this process.

In 2009 in Sweden, politicians at the Uppsala County Council and the director of the Uppsala University Hospital announced that visiting times in the ED had to be shortened, for quality and safety reasons. The 4-hour target was introduced. A financial incentive and time frame were also connected to this target, stating that by the end of the first six-month period 80% of patients had to be discharged or admitted to a ward within the 4-hour target. By the end of the year, the target had to be met for 100% of the patients. In order to meet this target, it was determined that teamwork would be introduced in the section of internal medicine in the ED at Uppsala University Hospital. The internal medical section was chosen because of an extraordinary increase in the number of patients and long visit times. Besides improving on meeting the 4-hour target, the goal of the introduction of teamwork was to involve the physician earlier in the ED care process, and to improve and secure the communication between healthcare professionals. The aim of the present study was to investigate the effects of teamwork in a Swedish emergency department on lead times and patient flow.

## Methods

### Study design

This study is part of a research project investigating teamwork in an ED. The research project is a longitudinal non-randomized intervention study with a mixed-method design, [13] to illuminate what makes team work [14] and to evaluate its effects from a quality and safety perspective.

**Figure 1:**
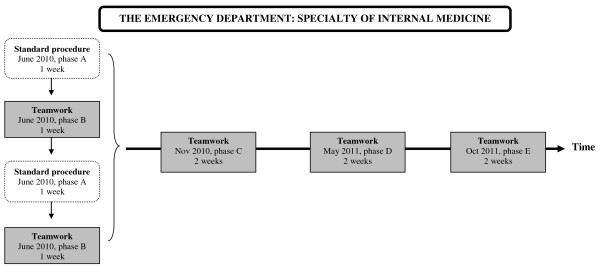
Description of study design and data collection periods.

A design with ABAB phases was first used, where A was the control phase (standard procedure) and B was the intervention phase. In addition, three follow-ups were conducted five (phase C), 11 (phase D) and 16 months (phase E) later, with each phase consisting of two weeks of data collection (see Figure 1 for more details). The recommendation for studying processes is that follow-ups over time are highly necessary to assure efficiency but still retain high quality of care and patient safety [15].

Mainly, the times of the follow-ups were based on: phase C) teamwork had been fully introduced in the section of internal medicine; phase D) 1-year follow-up after phase ABAB and phase E) 1-year follow-up after phase C.

### Setting and staff resources

An adult ED of a Swedish university hospital was the setting of the study. The university hospital covers a healthcare region of 1,966,504 inhabitants and the ED has a yearly visit level of more than 55,000 patients. The hospital is ranked as a Level 1 trauma hospital. The ED is divided into three medical sections: internal medicine, general surgery and orthopaedic surgery. On average, almost 60% of patient visits are registered in the section of internal medicine.

In total, about 120 registered nurses (RN) and assistant nurses (AN) are employed at the hospital and work in the ED. However, physicians are employed in the hospital in their medical specialty and the specialties are scheduled to provide the ED with physicians being on call. Thus, the number of on calls for each physician can vary between several on calls per month to a couple of on calls per year. A total of about 200 physicians can be on call in the ED. Work shifts are mainly divided into “Day” (about 7 am-4 pm), “Evening” (about 1 pm-9 pm) and “Night” (about 9 pm-7 am).

Depending on the work shift one or two physicians, one or two RNs and one or two ANs work together in each section in the ED. In addition, two acute teams work in the emergency room taking care of patients in immediate need of care (triage level RED). Each of these teams consists of one RN and one AN. During the shifts, one consultant was available for supporting the junior doctors.

Most of the RNs do not have a postgraduate exam, and the levels of the physicians’ competence vary greatly from intern to consultant. The majority of the physicians were junior doctors. At the time course for the study, none of the physicians were specially trained in emergency medicine. Over time, a limited number of physicians attended training for specialists in emergency medicine. However, none of them completed their specialist training during the data collection periods.

### Standard procedure

Before the intervention the work followed a traditional way of taking care of patients in the ED, where the RN, who allocated patients among the physicians, led the organizational work. According to the standard procedure patients met at least two nurses before seeing a physician. The physicians worked with any RN available, and all staff was, more or less, involved in every patient’s care processes within their specialty since the work process was arranged around tasks rather than patients. The nurses’ work station was placed in the middle of the ED and the physicians had their own offices in other places in the ED.

### Intervention

The intervention was the introduction of multi-professional teams by reorganization of the work processes. Each team consisted of one physician, one RN and one AN. The work process was arranged around the patients and each patient was handled by only one team through the whole care process. After the first assessment, the physician made a preliminary plan for further care in the ED, which was communicated to all members of the team and to the patient. The team members then worked in parallel but with ongoing communication and back-checking. Each team shared an office placed nearby the team’s examination rooms.

Teamwork was first implemented 8 am to 9 pm, weekdays only. Daytime, there were up to four different teams working in the section of internal medicine. At the third follow-up, one year after the implementation of the new working routine, teamwork had been implemented 24 hours a day, seven days a week.

Before teamwork was introduced, all staff members took part in an information meeting where the rationale for the new work process was laid out and implications for the professional roles were discussed. A handbook describing the work routine in detail was distributed to all physicians and nurses along with a flashcard, which highlighted the key team behaviours. Five months after the implementation of teamwork, an additional information meeting was conducted to involve new staff members and refresh staff members in the new work routine and to give and receive feedback to improve the implementation process and outcome.

No increase in number of staff was expected to be needed. However, a coordinator who allocated patients to the different teams as well as a facilitator who facilitated the implementation was introduced later.

**Figure 2:**
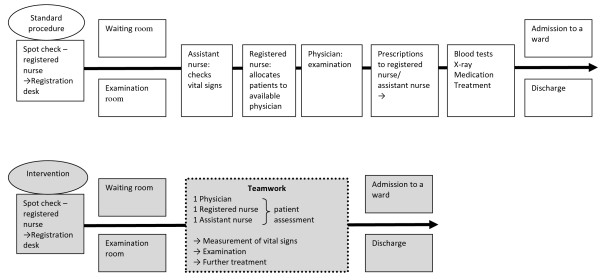
The process of care in the emergency department: intervention procedure versus standard procedure.

The differences between standard procedure and the intervention are further described in detail in Figure 2.

### Data collection

Data were collected from the electronic medical records, combined with data from the electronic tracking system. In the electronic tracking system, the following main variables were documented: main reason for seeking care, weekday/weekend day, time of day, number of patients (in total/at each section), mode of arrival, total visit time and outcome. Total visit time was calculated as time from registration at the registration desk to time of discharge, i.e. leaving the ED.

The research project was approved by the Regional Ethical Review Board in Uppsala (dnr 2010/170). Moreover, the Declaration of Helsinki and national guidelines for research were taken into consideration [16-18]. The electronic data were de-identified, and all stages of the process were handled in a confidential manner. None of the researchers were engaged in the clinical work at the section of internal medicine at the time of the data collection periods.

### Outcome measures

The primary outcome measures were as follows: a) number of patients handled within teamwork time, b) time to physician, c) total visit time and d) number of patients handled within the 4-hour target.

### Data analysis

Data from the electronic medical records and the tracking system were converted from excel data sheets to the statistical software package IBM SPSS, version 19 for analysis. Descriptive statistics are presented in medians, ranges, frequencies and percentages. To compare the different data collection periods according to outcome variables as well as background and visit characteristics, data were analyzed using non-parametric (Chi-2 test, Mann–Whitney U-test, Kruskal Wallis test) statistical tests. Non-parametric analyses were used due to skewed data. The P-value was set to 0.05. To adjust for potential confounders (gender, age and arrival mode), Willett’s residual method [19] for fully non-parametric adjustments has been used for the time variables and for the variable ”within 4 hours” we made adjustments with a multiple logistic regression model when enough data were provided. More than 30 reasons for seeking care in the ED were documented, and therefore, could this variable not be adjusted.

In the data analysis for outcome measures a) we used patient visits with arrival and discharge during the teamwork time (i.e. between 8 am and 9 pm, Monday to Friday).

Additionally, for outcome measures a), b), c) and d), we used patient visits with registered arrival between 8 am and 5 pm and discharge before 9 pm, i.e. patients that who could possibly be handled within the 4-hour target and within the time of the intervention (teamwork).

To be able to compare data from the initial ABAB design with those from the follow-ups, the A weeks were compiled into one group (phase A) and the B weeks into a group called phase B.

## Results

### Characteristics of study subjects

Within the time of day (8 am to 9 pm) when teamwork was implemented, a total number of 2,562 patients were registered as arriving in the section of internal medicine in the ED during the ten weeks of data collection. Of these, 48% (n = 1231) were men and 52% (n = 1331) were women. Median age was 64 years (range 17–99; interquartile range 33).

A total of 53 different reasons for seeking care were registered, whereby the majority of patients sought care for chest pain (n = 402; 22%). Almost half of the patients (n = 892; 49%) arrived by their own means, compared to 520 patients (28%) who arrived by ambulance and 18 patients (1%) who arrived by another form of transportation. Most of the patient visits (n = 1281; 50%) were registered as discharged to home, and 43% (n = 1106) were registered as admission to a ward. There were no statistical significances in background characteristics of the study subjects between the data collection periods.

There was some variation in number of patients for each intervention period: n = 458 (phase B), n = 536 (phase C), n = 514 (phase D) and n = 547 (phase E), compared to 507 patients in the control phase (phase A). A number of patients (n = 724) who arrived during teamwork time were discharged after the teamwork time was finished (i.e. after 9 pm): n = 124 (phase B; 27%), n = 152 (phase C; 28%), n = 170 (phase D; 33%) and n = 130 (phase E; 24%) compared to 148 (29%) patients in the control phase (phase A).

### Patients handled within teamwork time

Of the 2,562 patients, 1,838 (72%) were registered and discharged at the section of internal medicine before 9 pm. Forty-eight percent (n = 887) of these patients were men and 52% (n = 951) were women. Median age was 65 years (range 17–99 years; interquartile range 31). For each data collection period, the number of those handled within teamwork time (8 am-9 pm) was: n = 334 (phase B; 73%); n = 384 (phase C; 72%); n = 344 (phase D; 67%) and n = 417 (phase E; 76%), compared to n = 359 during the control phase (phase A; 71%).

Of the patients who could actually be handled within the 4-hour target, i.e. arrived before 5 pm and discharged before 9 pm (n = 1728), 842 (49%) were men and 886 (51%) were women. Median age was 65 years (range 17–99 years; interquartile range 31). The distribution of patient visits between the different data collection periods showed a small, non-significant increase in the number of patient visits handled within this time frame (p = 0.192) (Phase B n = 310 (68%); phase C n = 366 (68%); phase D n = 326 (63%); phase E n = 384 (70%) and phase A n = 342 (67%)).

### Time to physician

**Figure 3:**
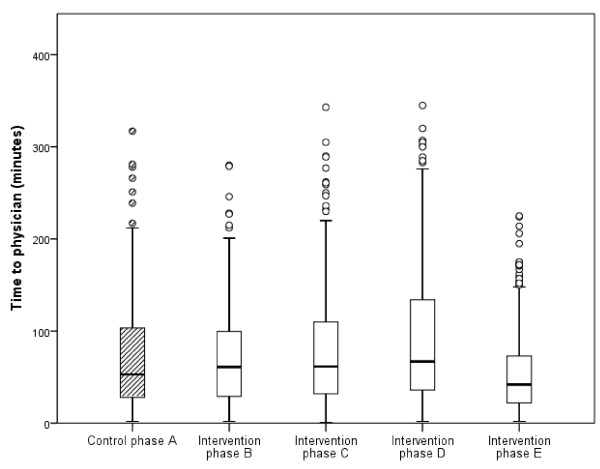
**The differences in time to physician between the control phase and the four intervention phases (phases B-E).** Phase E vs. phase A: p < 0.001.

Before teamwork was introduced (during the control phase, A), the median time to physician was 53 minutes (range 2–317 minutes; interquartile range 76). At the last follow-up (phase E), the median time to physician was 42 minutes (range 2–309 minutes; interquartile range 52). The difference between phases A and E was significant (*U* 52129.5; n_1_ = 375; n_2_ = 332; p < 0.001, adjusted for possible confounders p = 0.0005). However, there was also a difference in the opposite direction between phases A and D (the 11-month follow-up), when the median time for time to physician was 67 minutes (range 2–345 minutes; interquartile range 99; *U* 43614; n_1_ = 305; n_2_ = 332; p = 0.002, adjusted for possible confounders p = 0.0516). Differences in time to physician between phase A and phases B and C were not significant. Box plot for time to physician for each phase is presented in Figure 3.

### Total visit time

**Figure 4:**
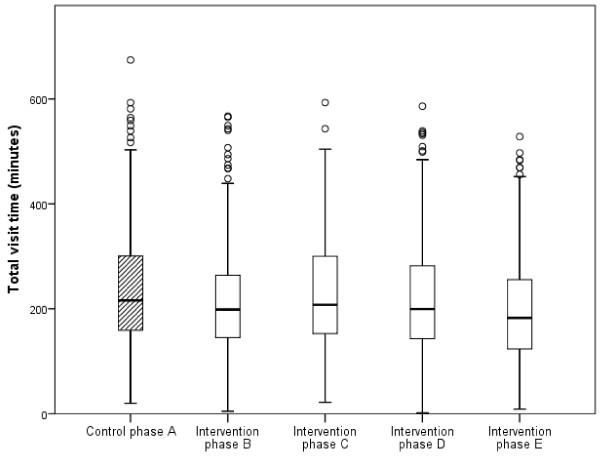
**The differences in total visit time between the control phase (phase A) and the four intervention phases (phases B-E).** Phase E vs. phase A: p < 0.001.

Total visit time in the ED for patients arriving between 8 am and 5 pm and being discharged before 9 pm varied between 2 and 674 minutes (n = 1728; median 199 minutes, interquartile range 138) during the data collection periods. The results showed significantly shorter total visit times for one teamwork period compared to the control phase (phase A): phase E (*U* 51284.5; n_1_ = 384; n_2_ = 342; p < 0.001; adjusted for possible confounders p = <0.0001; 39 minutes shorter on average). In comparing phase B (immediately after teamwork was introduced) with phase A, a significant reduction was seen (*U* 46522.0; n_1_ = 287; n_2_ = 342; p = 0.007; 17 minutes shorter on average), however, when adjusted for possible confounders no significance could be detected (p = 0.4837) (Figure 4).

### The 4-hour target

**Figure 5:**
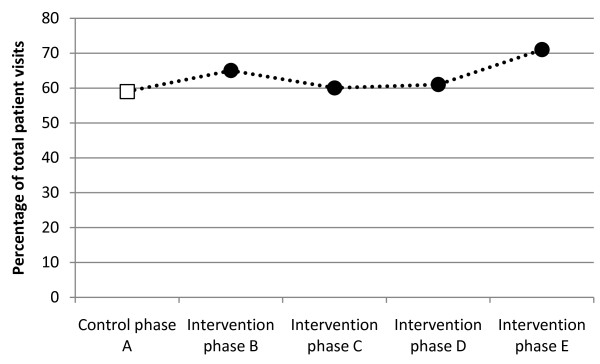
**Percentage of patient visits meeting the 4-hour target during the data collection periods.** Phase E vs. phase A: p = 0.002.

The frequency of patient visits that met the 4-hour target had increased at the last follow-up (phase E), and significant differences could be seen in this phase compared to the control phase (phase A) (χ2 16.518; df 4; p = 0.002, adjusted for possible confounders p = 0.0005). In phase E, 71% (n = 273) of the patient visits were handled within the 4-hour target compared to 59% (n = 200) in the control phase (phase A). In phases B, C and D, the frequency of patients handled within the 4-hour target varied between 60 and 65% (Figure 5).

## Discussion

In this article, we show that teamwork can contribute to a small but significant decrease in wasted time for patients in the ED, in terms of shorter time to physician and shorter total visit time. This effect of multi-professional teamwork on time to physician and total time in the ED is in line with a recent systematic review of interventions aiming at improving processes and patient flow in the ED [9]. Excessive total time in the ED has been linked to poor outcome, and has been suggested as an important quality indicator [20]. Time to physician, as well, has been described as an indicator of quality and safety in the ED, since seeing a decision-maker is a necessity in order to get a prompt assessment and, subsequently, adequate treatment [20]. Hence, our results indicate that teamwork may have a positive impact on these important quality indicators in the ED.

Furthermore, we show that the 4-hour target was met for a larger group of the patients in the ED at the last follow-up. This confirms the findings reported by Mason [21], who also showed that the introduction of a time target, in itself, was effective in reducing the proportion of patients staying in the ED longer than four hours [20]. However, the 4-hour target has also been criticized [22]. It is emphasized that lead times in the ED are not fully within the control of the ED [23]. For example, ED processes are reliant on other diagnostic facilities at the hospital such as radiology and laboratory, and not least the availability of hospital beds and the communication between wards and the ED. Hence, in the context of both ED development and improved patient flows, processes and safety, it is necessary to view ED together with and in interaction with the whole hospital [24]. This interdependence between the ED and actors outside the ED may be one reason why the effect of teamwork on lead times and the 4-hour target was limited in this study, and did not reach the goal set by the politicians.

It is important to note that the effect on lead times in this study was only evident at the last follow-up, almost 1.5 years after the introduction of teamwork. One interpretation may be that the chosen intervention was ineffective in achieving teamwork; or that it was effective, but that the resulting teamwork was not sufficiently linked to the outcomes. Another interpretation, which is consistent with other studies of improvement processes, is that change takes time. This is particularly true when it is a multi-faceted change in a complex environment with many different staff members involved. The implementation of change is difficult for several reasons: it is hard to reach staff with information, changes interfere with the hierarchies within hospitals, and achieving behavioural changes is difficult when there is a large and heterogenic group of staff who all need to change their way of working and do not always clearly see the benefits of the change right away [25,26]. Because of the physicians’ variety in number of on calls, we may also assume that time to adjust to the new work process would take longer than in contexts only dealing with permanent staff members. Also, over a period of time, changes in context often occur that may interfere with the implementation. In this particular case, organizational change and changes in managerial positions took place during the first follow-up (phase C) and were followed by a high turnover rate among RNs. This highlights that things outside the scope of the intervention may affect the interpretation of the results. It also indicates the usefulness of several follow-up periods. It is a challenge to investigate changes in clinical practice, but it is necessary in order to find efficient work procedures that lead to quality and safety improvements for patients and staff.

A wider question is whether the statistical significant effect on lead times also has clinical significance. In terms of patient safety, this is complicated since it is likely that the clinical consequences of long lead times differ between patients, depending on the severity of their medical condition. An endpoint to study would then be to pair lead times with triage levels. However, beyond patient safety issues, waiting times are also highly relevant from a patient satisfaction perspective in that long waiting times negatively influence patient satisfaction [27,28]. Thus, shortening lead times may nevertheless be important from this perspective.

### Limitations

This study was set in the ED’s section of internal medicine. Therefore, generalization to other sectors and specialties in healthcare may be limited to those parts that involve a large staff, physicians who are on call rather than part of the regular staff, and high dependability on other facilities at the hospital. However, the outcome in terms of lead times may be relevant for emergency care as the time the care process takes not only affects internal efficiency but also clinical outcome and patient safety in EDs.

Since the data are based on a number of two-week periods a number of months apart, it cannot be dismissed that the differences between the control phase and the follow-up phases were related to things other than teamwork. One factor that may contribute to a false effect of an intervention is the fact that when investigating change in clinical practice, the staff is not blinded to the fact that their work is being evaluated. Hence, it cannot be dismissed that staff may have behaved differently because they knew they were under study. However, it is unlikely that this would result in better results at the last follow-up. Another factor could be the variable levels of competence among the physicians, although there was no systematic difference in this respect between data collection periods. Furthermore, the large turnover rate among RNs means that, to some extent, there were different individuals on staff between the follow-up periods. Based on the importance of work experience in working in the ED, it is unlikely that having less experienced staff would result in better lead times. Rather, the fact that a new work routine and shorter lead times were achieved despite high turnover implies that focusing on changing work processes may be a way to implement teamwork.

In our study we used registry data whereby the time is manually registered when a patient physically leaves the ED. However, when a patient is admitted to the hospital there is often a delay from the moment when the admission decision is made and the time point at which there is a hospital bed available. This means that the lead times in this study are inflated. On the other hand, there is no evidence to suggest that there were any systematic differences between the measurement phases in this respect, and therefore the comparison over time is not likely to be affected. For the same reason the data regarding arrival mode had different missing values within the different data collection periods. There could be a possibility that patients arriving by ambulance would have been taken cared of in a shorter time, however, standard triage assessment were conducted and therefore the arrival mode unlikely could affect our main findings. Additionally, the findings from the adjustment for confounders should therefore be considered carefully, as it could be a result of limited data rather than effective adjustment.

## Conclusions

Teamwork, i.e. working in multi-professional teams, seems to contribute to quality improvement in emergency care in terms of small but significant decreases in lead times. This can be interpreted as an increased internal efficiency in the ED, e.g. minimised time waste. Additionally, the introduction of teamwork and subsequent reorganisation of the care process may further have improved patient safety by fostering a safer and accurate communication between staff members, and between staff members and patients. However, although efficient work processes like teamwork are necessary to ensure the safe handling of patients, they are likely not sufficient for achieving larger decreases in lead times or for meeting the 4-hour target. Rather, this may require the engagement of the whole hospital. Then, although the evaluation of clinical practice and complex change is difficult, it is important to evaluate new interventions both to ensure quality and the safety of patients, and to motivate staff to be engaged in the continuous improvement work of care.

## Competing interests

The authors declare that they have no competing interests.

## Authors’ contributions

The present study is a part of a research project investigating teamwork in the emergency department. A multidisciplinary research group has been formed, with members representing academic, as well as clinic. The authors’ contributions are as follow: AMA, UvTS and NF participated in the initiative of the research project and developed the study design. AMA participated in the data collection, performed the literature search, analysed the data and wrote the initial draft. UvTS performed the literature search, assisted in the data analysis and provided critical revision to the manuscript. NF supported the clinicians and the managers during the implementation of the intervention and contributed to the drafting of the manuscript and provided critical revision to the manuscript. All authors read and approved the final manuscript.

## Authors’ information

AMA: PhD, adj Senior Lecturer, Postdoctoral Researcher, RN, Clin Nurs Spec (Emerg Care).

UvTS: Associate Professor, PhD, Reg. Psych.

NF: PhD, MD, Head of Department of Emergency Care, Sodersjukhuset.

## References

[B1] IOM - Institute of Medicine To Err Is Human. Building a Safer Health System 2000 Washington, D.C.: National Academy Press 25077248

[B2] Olshaker JS Managing emergency department overcrowding Emerg Med Clin North Am 2009 27 593 603 10.1016/j.emc.2009.07.004 19932394

[B3] Bernstein SL Asplin BR Emergency department crowding: old problem, new solutions Emerg Med Clin North Am 2006 24 821 37 10.1016/j.emc.2006.06.013 16982341

[B4] NHS - National Health Service, Department of Health The NHS Plan. A Plan for Investment. A Plan for Reform 2000 London, England: Stationery Office

[B5] Willoughby KA Chan BTB Strenger M Achieving wait time reduction in the emergency department Leadersh Health Serv 2010 23 304 19 10.1108/17511871011079010

[B6] Leonard MW Frankel AS Role of effective teamwork and communication in delivering safe, high-quality care Mt Sinai J Med 2011 78 820 6 10.1002/msj.20295 10.1002/msj.20295 22069205

[B7] Fernandez R Kozlowski SW Shapiro MJ Toward a definition of teamwork in emergency medicine Acad Emerg Med 2008 15 1104 12 10.1111/j.1553-2712.2008.00250.x 18828831

[B8] Webster JS King HB Toomey LM Henriksen K, Battles JB, Keyes MA, Grady ML Understanding quality and safety problems in the ambulatory environment: seeking improvement with promising teamwork tools and strategies Advances in Patient Safety: New Directions and Alternative Approaches (Vol. 3: Performance and Tools) 2008 Rockville (MD): Agency for Healthcare Research and Quality (US) 1 15 21249939

[B9] Oredsson S Jonsson H Rognes J A systematic review of triage-related interventions to improve patient flow in emergency departments Scand J Trauma Resusc Emerg Med 2011 19 19:43 10.1186/1757-7241-19-43 PMC3152510 21771339

[B10] Eitel DR Rudkin SE Malvehy MA Improving service quality by understanding emergency department flow: a white paper and position statement prepared for the American academy of emergency medicine J Emerg Med 2010 38 70 79 10.1016/j.jemermed.2008.03.038 18514465

[B11] Kozlowski SWJ Bell BS Borman WC, Ilgen DR, Klimoski RJ Work groups and teams in organizations Handbook of psychology (Vol. 12): Industrial and Organizational Psychology 2003 New York: Wiley 333 375

[B12] Hackman JR Lorsch J The design of work teams Handbook of organizational behavior 1987 Englewood Cliffs, NJ: Prentice-Hall

[B13] Des Jarlais DC Lyles C Crepaz N TREND Group Improving the reporting quality of nonrandomized evaluations of behavioral and public health interventions: the TREND statement Am J Public Health 2004 94 361 366 10.2105/AJPH.94.3.361 14998794 PMC1448256

[B14] Mazzocato P Hvitfeldt Forsberg H Thiele Schwarz U Team behaviors in emergency care: a qualitative study using behavior analysis of what makes team work Scand J Trauma Resusc Emerg 2011 15 19:70 10.1186/1757-7241-19-70 PMC3248857 22085585

[B15] Salas E Sims DE Burke CS Is there a ''Big Five'' in teamwork? Small Group Research 2005 36 555 10.1177/1046496405277134

[B16] World Medical Association [Internet]. France: World Medical Association, Inc.; [cited 2012Feb22] World Medical Association Declaration of Helsinki - Ethical Principles for Medical Research Involving Human Subjects Available from: http://www.wma.net/en/30publications/10policies/b3/index.html 19886379

[B17] SFS 2003:460. Lag om etikprövning av forskning som avser människor. [Act concerning the Ethical Review of Research Involving Humans] [Internet]. Stockholm: Swedish Code of Statutes [cited 2012Feb 22] Available from: http://www.notisum.se/rnp/SLS/LAG/20030460.htm. In Swedish

[B18] CODEX Rules and guidelines for research [homepage on the Internet]. Uppsala, Sweden: Swedish Research Council in cooperation with the Centre for Research Ethics & Bioethics at Uppsala University. [updated 2009 March 27; cited 2012 Feb 22] Available from: http://www.codex.uu.se/

[B19] Willett W Stampfer MJ Total energy intake: implications for epidemiologic analyses Am J Epidemiol 1986 124 17 27 3521261 10.1093/oxfordjournals.aje.a114366

[B20] Heyworth J Emergency medicine-quality indicators: the United Kingdom perspective Acad Emerg Med 2011 18 1239 41 doi:10.1111/j.1553-2712.2011.01223.x 10.1111/j.1553-2712.2011.01223.x 22168185

[B21] Mason S Keynote address: United Kingdom experiences of evaluating performance and quality in emergency medicine Acad Emerg Med 2011 18 1234 8 doi:10.1111/j.1553-2712.2011.01237.x 10.1111/j.1553-2712.2011.01237.x 22168184

[B22] Mortimore A Cooper S The “4-hour target”: emergency nurses’ views Emerg Med J 2007 24 402 4 10.1136/emj.2006.044933 17513535 PMC2658273

[B23] Weber EJ Mason S Carter A Emptying the corridors of shame: organizational lessons from England's 4-hour emergency throughput target Ann Emerg Med 2011 57 79 88 e1 10.1016/j.annemergmed.2010.08.013 21251521

[B24] Pines JM Hilton JA Weber EJ International perspectives on emergency department crowding Acad Emerg Med 2011 18 1358 70 10.1111/j.1553-2712.2011.01235.x 10.1111/j.1553-2712.2011.01235.x 22168200

[B25] Hill M Hupe PL Implementing Public Policy: Governance in Theory and Practice 2002 London: Sage

[B26] Grol R Baker R Moss F Quality improvement research: understanding the science of change in health care, volume 11 2004 London: BMJ Books 10.1136/qhc.11.2.110 PMC1743590 12448794

[B27] Dinh MM Enright N Walker A Parameswaran A Chu M Determinants of patient satisfaction in an Australian emergency department fast-tracksetting Emerg Med J 2012 Epub ahead of print 10.1136/emermed-2012-201711 23139091

[B28] Pines JM Iyer S Disbot M Hollander JE Shofer FS Datner EM The effect of emergency department crowding on patient satisfaction for admitted patients Acad Emerg Med 2008 15 9 825 831 10.1111/j.1553-2712.2008.00200.x 19244633

